# The Voluntary Hospitals Commission

**Published:** 1921-09-03

**Authors:** 


### The Voluntary Hospitals Commission.

### ITS CHAIRMAN'S VIEWS ON LOCAL COMMITTEES.

The Earl of Onslow, Chairman of the Voluntary Hospitals' Commission, has given an interview to the *Surrey Advertiser* on the work of the new body, from which we take the principal points. We deal editorially with the subject on p. 381.

Lord Onslow explained that in his opinion the most important function of the Commission would be to help the hospitals to evolve a system whereby they might maintain themselves. It has, therefore, taken steps to form local committees throughout the country. The method suggested for these may be recalled. The county council will choose two, the town council one; the larger hospitals one, the smaller hospitals one; the medical profession in the county will choose one consultant and one general practitioner. To these six members will be added one from each county borough. The Commission will appoint not more than five members from residents in the county, who will therefore be in a minority to those appointed locally. Lord Onslow added that "a large field of experience, insufficiently used, exists among those ladies who were commandants of hospitals during the war." Much is to be hoped from the first-hand experience thus obtained, especially by the ladies entrusted with the charge of large primary hospitals, similar in many ways to the average general hospitals of 100 beds.

Dealing with the shortage of hospital beds, and explaining that the Government was unable to make any grants for extension of buildings, Lord Onslow recalled his recent speech in the House of Lords. He again urged that some scheme might be evolved to make use of the number of constantly vacant beds in Poor-Law infirmaries.

He hopes also that the county committees will co-ordinate appeals, and emphasised the value of regular and systematic contributions from employers and employed.

The Commission is suggesting the adoption of a uniform system of accounts. Schemes for co-operative purchase and the transfer of patients might also be devised.

The local committees, Lord Onslow explained, are not expected to control the hospitals, but to assist and advise them; and those solvent hospitals which may feel that the help of the local committees is superfluous should remember that the voluntary system cannot be saved without the co-operation and mutual assistance of all institutions and persons connected with it. Therefore the solvent institutions must co-operate with the less prosperous, though funds, of course, will not be transferred or the property of individual hospitals interfered with.

In principle, convalescent homes were outside the scope of the Commission, but Lord Onslow explained that they can be granted financial assistance if it is considered that any particular institution is helping to increase the hospital accommodation in the area. The Commission will rely largely on the recommendation of the local committees.

